# PERSONALIZED MUSIC PLAYLISTS FOR HOSPITALIZED OLDER ADULTS: CREATING AND REVISING VOLUNTEER WORKFLOW

**DOI:** 10.1093/geroni/igae098.2469

**Published:** 2024-12-31

**Authors:** Victor Nguyen, Angela Morka, Kayla Wong, Maanasa Nandigam, Eliza Kiwak, Judy Li, Jody Sharninghausen, Richard Marottoli

**Affiliations:** Yale University, New Haven, Connecticut, United States; Yale University, New Haven, Connecticut, United States; Yale University, New Haven, Connecticut, United States; Yale University, New Haven, Connecticut, United States; Yale University School of Medicine, New Haven, Connecticut, United States; Yale University School of Medicine, New Haven, Connecticut, United States; Yale University School of Medicine, New Haven, Connecticut, United States; Yale University School of Medicine, New Haven, Connecticut, United States

## Abstract

Personalized music has been shown to decrease the incidence of neuropsychiatric symptoms among hospitalized older adults. As part of the “Creativity in Aging” project at Yale Geriatrics, this quality improvement study aims to evaluate the process and feasibility of a personalized music program in an inpatient geriatrics unit. We collaborated with the hospital volunteer department to credential and train five undergraduate volunteers in building personalized music playlists for hospitalized older adults to listen to during their stay. From October 2023 to February 2024, 88 patients were referred to the program by hospital staff, and 57 patients participated. Here we describe the process of developing and revising the pilot. Following “test” sessions led by a medical student and resident physician familiar with the unit, an initial protocol was developed. Volunteers used REDCap and paper spreadsheets to organize HIPAA-secure patient data including referrals, linkage to personalized playlists stored on iPads, and brief pre- and post-listening surveys. Volunteers filled out feedback forms after each weekly shift to identify workflow issues and suggest improvements. Challenges included 1) identification of appropriate patients and 2) hand-off of patient referrals, notes, and next steps between shifts. Consultation with multiple stakeholders including volunteers, nursing managers, and attending physicians, led to solutions: 1) recruitment of nursing staff and creation of resident physician training module, after which nurses and resident physicians became key patient referral sources, and 2) remodeling of REDCap to manage volunteer “hand-off” communication, replacing the physical note-keeping system. We aim to make this a sustainable volunteer program.

